# Quorum sensing of microalgae associated marine *Ponticoccus* sp. PD-2 and its algicidal function regulation

**DOI:** 10.1186/s13568-017-0357-6

**Published:** 2017-03-09

**Authors:** Wendan Chi, Li Zheng, Changfei He, Bin Han, Minggang Zheng, Wei Gao, Chengjun Sun, Gefei Zhou, Xiangxing Gao

**Affiliations:** 1 Key Laboratory for Marine Bioactive Substances and Modern Analytical Technology of the First Institute of Oceanography, State Oceanic Administration, No. 6 Xianxialing Road, Qingdao, 266061 Shandong People’s Republic of China; 2 Laboratory for Marine Ecology and Environmental Science, Qingdao National Laboratory for Marine Science and Technology, Qingdao, 266071 People’s Republic of China; 3 School of Life Science of Yantai University, Yantai, 264000 People’s Republic of China; 4 National Deep Sea Center, State Oceanic Administration of China, Qingdao, 266237 People’s Republic of China

**Keywords:** AHLs, Algicidal activity, β-Cyclodextrin, Microalgae-associated bacteria, Quorum sensing

## Abstract

Quorum sensing (QS) systems play important roles in regulating many physiological functions of microorganisms, such as biofilm formation, bioluminescence, and antibiotic production. One marine algicidal bacterium, *Ponticoccus* sp. PD-2, was isolated from the microalga *Prorocentrum donghaiense*, and its *N*-acyl-homoserine lactone (AHL)-mediated QS system was verified. In this study, we analyzed the AHLs profile of strain PD-2. Two AHLs, 3-oxo-C8-HSL and 3-oxo-C10-HSL, were detected using a biosensor overlay assay and GC–MS methods. Two complete AHL-QS systems (designated *zlaI*/*R* and *zlbI*/*R*) were identified in the genome of strain PD-2. When expressed in *Escherichia coli*, both *zlaI* and *zlbI* genes could each produce 3-oxo-C8-HSL and 3-oxo-C10-HSL. Algicidal activity was investigated by evaluating the inhibitory rate (IR) of microalgae growth by measuring the fluorescence of viable cells. We found that the metabolites of strain PD-2 had algicidal activity against its host *P. donghaiense* (IR 84.81%) and two other red tide microalgae, *Phaeocystis globosa* (IR 78.91%) and *Alexandrium tamarense* (IR 67.14%). β-cyclodextrin which binds to AHLs and inhibits the QS system reduced the algicidal activity more than 50%. This indicates that inhibiting the QS system may affect the algicidal metabolites production of strain PD-2. Our study indicated that a QS-regulated algicidal system may play a potential role in the process of red tides disintegration. QS might be a potential way to control red tides.

**Electronic supplementary material:**

The online version of this article (doi:10.1186/s13568-017-0357-6) contains supplementary material, which is available to authorized users.

## Introduction

Quorum sensing (QS) is a cell density-dependent system for information transfer among bacteria. It enables the bacterial cells to sense changes in cell density through the concentration of signal molecules called autoinducers (AI) released by bacteria themselves (Guo et al. 2011). When the bacteria grow to a high cell density, the concentration of AI could reach a threshold and bind specifically to a receptor protein. Then, the complex will activate the transcription of target genes and regulate specific functions including biofilm formation, bioluminescence, the secretion of virulence factors, and sporulation (Davies et al. 1998; Greenberg et al. 1979; Nealson et al. 1970). In addition to directly affecting gene regulation, increasing evidences show that cross-kingdom QS signaling between associated bacteria and their hosts also confers other ecological functions indirectly, such as growth-retarding effect of the rotifer (Gambello and Iglewski 1991), the settlement of seaweed zoospores (Joint et al. 2007), and carospore liberation of red macroalgae (Singh et al. 2015). Therefore, QS systems and their regulation functions in bacteria have become hot topics in the fields of marine environment, aquaculture, medicine, and bioengineering.

To date, the most studied intercellular AI molecules are *N*-acyl-homoserine lactones (AHLs), which can be detected by biosensors, gas chromatography–mass spectrometer (GC–MS), and high-performance liquid chromatography-mass spectrometer (HPLC-MS) (Li et al. 2015). These AHLs are generated by an autoinducer synthase, the product of a *luxI* type gene, and the receptor is a cognate luxR type protein. Though there is an increasing number of AHL synthetase genes with some conserved regions in the genomic databases,the overall similarity between luxI homologs from different genera is rather low (Fuqua et al. 1996). Therefore, it is necessary to determine the profiles of AHLs produced by different *luxI* homologs to better understand the QS system.

Red tides are natural phenomena that are caused by the explosive proliferation of microalgae (Guzmán et al. 2016). Most red tides result in serious environmental problems that are harmful or toxic to humans, fish, shellfish, marine mammals, and birds (Hu et al. 2016). The algicidal bacteria that are associated with red tide algae play an important role in controlling their host’s biomass. In the initial and developmental stages of red tides, algicidal bacteria show no algicidal activity. In the alage blooming stage, the growth of algicidal bacteria was promoted by the nutrients that are organic materials released by the algae. Subsequently, the bacteria begin to produce algicidal compounds that kill their host cells. It is interesting to find that some algicidal bacteria such as *Cytophaga* strains J18/M01 (Mirsutani et al. 1992) and A5Y (Imai et al. 1993), *Flavobacterium* strain 5N-3 (Imai et al. 1992), and *Flavobacteriaceae* strain S03 (Roth et al. 2007) must reach a cell density of at least 10^6^ cells mL^−1^ before they show any algicidal activity. Li et al. (2014) found that *Shewanella* sp. Lzh-2 must reach a bacterial cell density threshold of at least 10^8^ cells mL^−1^ before any algicidal compound can be detected. There are also reports that some microalgae could inhibit bacterial quorum sensing activity (Castang et al. 2004). Because algicidal activity is density-dependent, we hypothesize that the algicidal activity of the microalgae associated bacteria may be regulated by QS system and the interaction between microalgae and their associated algicidal bacteria could be mutually restrictive. This probably is an important factor for maintaining the dynamic balance of phycosphere.

It has been proven that QS systems in some bacteria can regulate the production of secondary metabolites, such as antibiotics, including algicidal compounds. One marine bacterium, MS-02-063, belonging to the *γ*-*proteobacteria*, produces a red, algicidal pigment (Nakashima et al. 2006). It is interesting to note that pigment production was also inhibited by adding β-cyclodextrin (β-CD), which forms a complex with AHLs in media, as a QS-quenching substance (Morohoshi et al. 2013). Although it was suggested that the algicidal pigment production was controlled by AHLs-mediated QS, this study neither confirmed the presence of a QS system at the genetic level nor did it find any AHLs molecules. In a subsequent research, Guo et al. (2016) found a bacterium *Aeromonas* sp.GLY-2107 which could produce two algicidal compounds against *Microcystis aeruginosa*. They proved that the production of the two algicidal compounds was controlled by QS system. However, this bacterium was screened from freshwater and was not associated with microalgae *M. aeruginosa*. To date, there has been no report on the QS regulated algicidal activity investigated through the interaction between microalgae and their associated bacteria.

In this work, The QS systems of *Ponticoccus* sp. PD-2, which is a marine microalgae-associated bacterium with algicidal activity, were confirmed by detecting AHL molecules and annotating luxI type autoinducer synthase and *luxR* type receptor genes. We analyzed the AHL synthetase functions by introducing *LuxI* homologes into *Escherichia coli*. The algicidal activity of strain PD-2 was evaluated and compared after adding β-CD into the culture medium. The purpose of this study was to understand AHL-based QS systems in *Ponticoccus* sp. PD-2, and to prove the hypothesis that the microalgae associated bacterium may kill the host through QS system. We expect to find a novel way to control red tides by biological treatment via enhancing QS activities.

## Materials and methods

### Strains and growth conditions

*Ponticoccus* sp. PD-2 used in this study was obtained from China General Microbiological Culture Collection Center (CGMCC 1.16027). It was isolated from *Prorocentrum donghaiense*. Strain PD-2 was grown in marine broth 2216E (Xu et al. 2012) at 27 °C with shaking at 150 rpm, unless otherwise indicated. *E. coli* BL21 (DE3) was used to propagate recombinant plasmids and to overexpress zlaI and zlbI proteins. For *E. coli* strains, the bacteria were grown in Luria–Bertani (LB) medium (Britstein et al. 2016) at 37 °C, with shaking at 150 rpm. Transformed cells were grown in LB broth supplemented with 100 μg mL^−1^ ampicillin (Sigma-Aldrich, St. Louis, MO, USA). The microalgae strains included red tide species *P. donghaiense* CCMM1007, *Phaeocystis globosa* CCMM5010, and *Alexandrium tamarense* CCMM1002 (CCMM: Culture Collection of Marine Microalgae). All the indicator microalgae strains were courtesy of the Institute of Oceanography, Chinese Academy of Science and deposited in CMBGCAS. f/2 medium (Guillard and Ryther 1962) was prepared with seawater collected from the Qingdao shore, and it was filtered through a 0.45-μm pore membrane. The medium was autoclaved at 121 °C for 20 min. The algae were cultured in f/2 medium at 25 °C under cool-white fluorescent lamps (100 μmol photons m^−2^ s^−1^) with a 12:12 light/dark cycle, with shaking three times per day.

The biosensor *Agrobacterium tumefaciens* KYC55 (pJZ372) (pJZ384, pJZ410), which was provided by Professor Hill at the Institute of Marine and Environmental Technology, University of Maryland, USA. The reporter strain KYC55 (Karina et al. 2013) was prepared by growing it in 1× ATGN minimal medium (Tempé et al. 1977) with 100 μg mL^−1^ spectinomycin dihydrochloride pentahydrate, 4.5 μg mL^−1^ tetracycline, and 100 μg mL^−1^ gentamicin at 30 °C, with shaking at 180 rpm. A subculture was made in the same medium with an initial 1:500 dilution of the cells. The subculture was grown under the same conditions and harvested when an optical density at 630 nm (OD_630_) reached 0.35. The cells were harvested by centrifugation at 15,000×*g* for 10 min. The cell pellet was suspended in sterile 30% glycerol to a calculated OD_630_ of 12. One-milliliter aliquots of the cells were stored at −80 °C.

### Chemicals

AHL standards (C6-HSL, 3-oxo-C6-HSL, C8-HSL, 3-oxo-C8-HSL and 3-oxo-C10-HSL) were purchased from the Cayman Chemical Company (Ann Arbor, Michigan, USA). Chromatographic-grade methanol and 99.9% ethyl acetate were purchased from Sigma-Aldrich (Buchs, Switzerland). The standards were dissolved in methanol at a concentration of 1 mM and stored at −20 °C.

### Extraction of metabolites of strain PD-2

Bacterial cell suspensions were centrifuged at 12,000 rpm for 5 min. One hundred and fifty milliliters of the cell-free supernatant solution was adjusted to pH 7 with 6 M HCl solution, preserved overnight at 4 °C, and then extracted according to the procedure described previously (Cataldi et al. 2004, 2007). Briefly, 150 mL of the cell-free supernatant solution was extracted twice with 150 mL of ethyl acetate each. The combined organic phases were evaporated by rotary evaporators at 37 °C. The residue was redissolved in 200 μL of ethyl acetate and stored at 4 °C.

### Analysis of the AHL profile

#### Bioautography analysis

All AHLs produced by strain PD-2 were screened using the *A. tumefaciens* KYC555-based biosensor system developed by Zhu et al. (2003). The reporter overlay was prepared by cooling 90 mL of 0.6% agar to 40 °C and adding 5 mL of 20× AT salts (15 mM (NH_4_)_2_SO_4_, 0.6 mM MgSO_4_·7H_2_O, 0.06 mM CaCl_2_·2H_2_O, and 0.0071 mM MnSO_4_·H_2_O), 5 mL of 20× AT buffer (79 mM KH_2_PO_4_, pH 7.0), 1 mL of 50% (w/w) glucose, 200 μL of 20 mg mL^−1^ X-gal, and 1 mL of the prepared the KYC55 cells. Extracts were spotted onto C18 reverse-phase thin-layer chromatography (TLC) plates (Mallinckrodt Baker, Philipsburg, NJ, USA) and developed with 60% methanol as the mobile phase. The TLC plates were dried, sterilized with ultraviolet light, and placed in an autoclaved Petri dish. The AHLs reporter overlay was poured on top of the plate. The plate was incubated at 30 °C overnight to allow the developing of blue colonies that indicate the existence of AHLs.

#### GC–MS analysis

The GC–MS analysis was performed according to Cataldi et al. (2007). The analysis of AHLs in the extracts from bacterial cultures was performed by a GC system that was interfaced to a single-quadrupole mass spectrometer (7980A/5975C, Agilent Technologies, Santa Clara, CA, USA). Samples were injected with the split mode (5:1) into an HP-5 MS capillary column, 30 m × 250 μm internal diameter and 0.25-μm-thick film coated with 5% PhMe siloxane. High-purity helium was used as carrier gas at a flow rate of 1 mL min^−1^. The GC injector temperature was set at 200 °C. The oven temperature program was optimized to hold at 150 °C for 3 min and then increase by 28 °C min^−1^ up to 280 °C. The transfer line temperature was adjusted to 280 °C. Mass spectrometry conditions were as follows: the electron ionization source was set to 70 eV; the emission current was 500 μA; the MS quad was set to 150 °C; and the MS source was set to 230 °C. The mass spectrometer was run both in full-scan mode (*m/z* 15–800) and the chromatography analysis performed in SIM mode (*m/z* = 143).

### Analysis of AHL synthases in strain PD-2

The genome sequence of strain PD-2 was sequenced and analyzed by Majorbio Co., Ltd. (Shanghai, China). The whole genome of strain PD-2 was submitted to GenBank (AWRV02000000) (Zheng et al. 2015). From the annotated sequences of *Ponticoccus* sp. PD-2, two clusters of *luxI* and *luxR* homologues were identified. After analyzing the candidate genes related to AHL production we found two AHL-sythase genes. The first AHL-sythase gene named *zlaI* is located in contig 600 with a length of 870 bp. The second AHL-synthase gene named *zlbI* is located in contig 616 with a length of 639 bp. The two AHL-related genes (*luxR* family transcriptional regulator), *zlaR* and *zlbR*, were located in the upstream position of *zlaI* and *zlbI*, respectively. Each cluster of the *luxI* and *luxR* genes is contiguous. The two QS (*luxI*/*R* homologs) genes were named *zlaI*/*R* and *zlbI*/*R* respectively. Comparative analysis of the *zlaI/R* and *zlbI/R* sequences and the closest related sequences were performed in the National Center for Biotechnology Information database (http://www.ncbi.nlm.nih.gov), and homologous proteins were retrieved using the protein Basic Local Alignment Search Tool (BLASTP). Sequences were aligned using DNASTAR.Lasergene.v7.1 software. A phylogenetic tree was constructed with MEGA 7.0 software (Kumar et al. 2016), using the neighbor-joining algorithm with 1000 permutations.

### DNA manipulations

The complete sequences of the *zlaI* and *zlbI* genes were amplified by polymerase chain reaction (PCR). Primers with the following sequences were used: zlaI-F (5′-CGGGATCCATGAATGGAGTGGTCAAAAT-3′), zlaI-R (5′-CCCTCGAGTCAGGCCCGCTTTGCCTTCA-3′), zlbI-F (5′-CGGGATCCATGATCCGCTACATCTACGG-3′), and zlb-R (5′-CCCTCGAGTCAGGCGGCAACCATCTGCT-3′). The 5′-end primer included a *Bam*HI restriction site, and the 3′-end primer included an *Xho*I restriction site. PCR was performed by incubation for 2 min at 95 °C, followed by 30 cycles of 30 s at 95 °C, 30 s at 60 °C, and 1 min at 72 °C, with a final elongation step of 10 min at 72 °C.

### Function determination

The amplified *zlaI* and *zlbI* genes were cloned into pGEX-6P-1, and the resulting recombinant plasmids were labeled as pGEX-*zlaI* and pGEX-*zlbI*, respectively. These two plasmids were transformed into *E. coli* BL21 (DE3). The sequence of *zlaI* and *zlbI* cloned into pGEX-6P-1 plasmid were verified by PCR with primers (F:5′-GGGCTGGCAAGCCACGTTTGGTG-3′; R:5′-CCGGGAGCTGCATGTGTCAGAGG-3′). Then, the transformants were grown on LB plate containing ampicillin (100 μg mL^−1^) at 37 °C for 24 h with shaking at 150 rpm. The AHLs production profiles of these two transformants were analyzed as described in the bioautography analysis section.

### Effects of β-CD on the growth of strain PD-2

The effects of β-CD on the growth of strain PD-2 were tested before determining the effects of β-CD on autoinducer activities. Strain PD-2 was inoculated into 2216E medium and incubated at 27 °C, with shaking at 150 rpm for 20 h. Then, the bacterial cultures were mixed with various concentrations of β-CD solutions (final concentrations of β-CD were 1, 2, and 5 mg mL^−1^ respectively) and continued incubation at 27 °C for another 64 h with shanking 150 rpm. Growth curves of the bacteria were plotted according to the periodically measured OD_630_. β-CD at a 1 mg mL^−1^ concentration, which had the smallest effects on growth, was selected for the subsequent algae-inhibition experiments to minimize the effects resulting from the addition of β-CD.

### Assessment of algicidal activity

The algicidal effect of strain PD-2 was assessed as described by Xu et al. (2012). The target species included *P. donghaiense*, *P. globosa,* and *A. tamarense.* The final concentrations of *P. donghaiense*, *P. globosa,* and *A. tamarense* were adjusted to 1.4 × 10^5^, 1.1 × 10^6^ and 2.3 × 10^4^ cells mL^−1^ at the exponential phase respectively. Strain PD-2 was grown for 20 and 84 h at 27 °C, with shaking at 150 rpm. Metabolites were extracted as described previously. The extracts of the culture were redissolved in dimethyl sulfoxide to a final concentration of 20 mg mL^−1^. Subsequently, 5 μL of the extracts was added to a 10 mL glass tube containing 5 mL of the algae cultures and incubated at 25 °C. Meanwhile, as a control, 5 μL of an extract of 22l6E medium was added to 5 mL of the algae cultures after 5 day of cocultivation. The algicidal activities were measured by the fluorescence method under the conditions of EX (excitation spectrum) = 463 nm/EM (emission spectrum) = 640 nm.

The inhibition rate (IR, %) was calculated based on the following equation:

$$\text{IR} = \frac{F_{\text{control}} - F_{\text{treatment}}}{F_{\text{control}}} \times {100}{\%}$$

where Ftreatment and Fcontrol represent the fluorescence values in the treatment and control, respectively.

### Investigation of the QS-mediated regulation of the algicidal activity of strain PD-2

To investigate whether QS regulates algicidal activity, strain PD-2 was initially grown for 20 h at 27 °C. Then β-CD was added to the culture and the culture was incubated for 64 h at 27 °C, with shaking at 150 rpm. Then, the metabolites were extracted to analyze changes in AHLs production and algicidal activity. The method of detecting the production of AHLs and algicidal activity based on bioautography analysis and assessment of algicidal activity respectively.

## Results

### Analysis of the AHL profile

#### Bioautography analysis

**Fig. 1:**
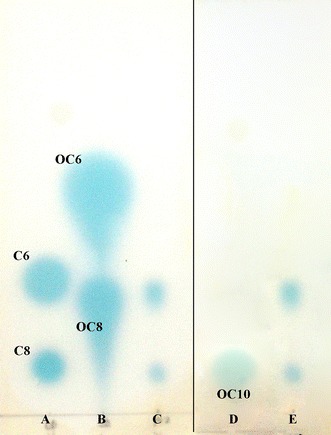
Profiles of AHLs produced by strain PD-2 based on bioautography assay. The biosensor is *Agrobacterium tumefaciens* KYC55. *Lane A* mixture of synthetic HSL standards: C6 (C6-HSL); C8 (C8-HSL). *Lane B* mixture of synthetic 3-oxo-HSL standards: 3-oxo-C6 (3-oxo-C6-HSL); 3-oxo-C8 (3-oxo-C8-HSL). *Lane D* OC10 (3-oxo-C10-HSL) standard. *Lane C* and *E* extracts of strain PD-2.There are two *blue spots* on the plate. This indicate two AHLs existed in strain PD-2. The *upper spot* matched 3-oxo-C8-HSL standard and the *lower spot* matched 3-oxo-C10-HSL standard

TLC overlay assay with the biosensor KYC55 suggested that strain PD-2 produced two AHLs (Fig. 1). The retardation factor (RF) values of lower spot and upper spot were 0.12 and 0.31 respectively. Five standards (C6, OC6, C8, OC8 and OC10) were shown in Fig. 1. According to the RF values of the AHLs, two AHLs matched standards, which were synthetic *N*-3-oxo-octanoyl-L-homoserine lactone (3-oxo-C8-HSL) and *N*-(3-oxodecanoyl)-L-homoserine lactone (3-oxo-C10-HSL). GC–MS experiments were performed subsequently to confirm the AHLs of strain PD-2.

#### GC-MS analysis

**Fig. 2:**
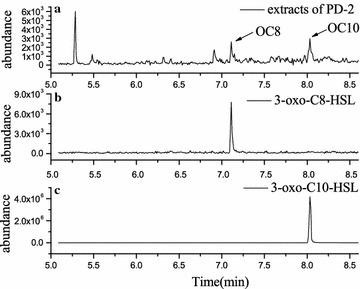
GC-MS chromatogram in SIM mode at *m/z*143. **a** Extract of strain PD-2. **b** 3-oxo-C8-HSL standard, RT = 7.111 min. **c** 3-oxo-C10-HSL standard, RT = 8.033 min

A chromatography analysis was performed in SIM mode using an ion with *m/z* = 143 as a marker fragment. The chromatography of the AHL standards were shown in Fig. 2b and c. Through analyzing the MS information, two peaks in PD-2 extracts showed in Fig. 2a of the retention times (RT) were consistent with the standard of 3-oxo-C8-HSL and 3-oxo-C10-HSL at 7.111 and 8.033 min.

### Comparative genomics and phylogenetic tree

**Fig. 3:**
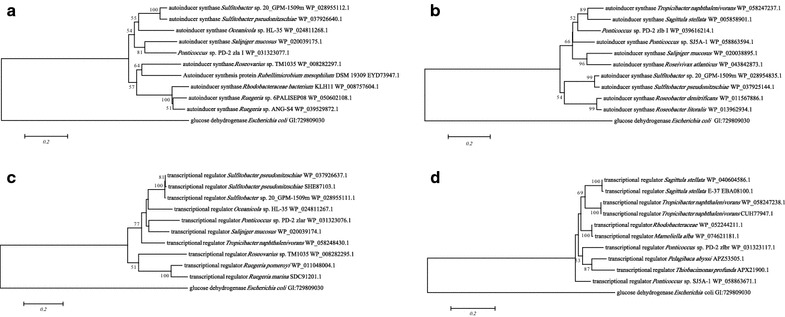
Phylogenetic tree showed the phylogenetic position of AHL synthases and transcriptional regulator of strain PD-2. The tree is drawn to scale, with branch lengths in the same units as those of the evolutionary distances used to infer from the phylogenetic tree. The *horizontal talbar at the bottom* represents evolutionary distance as 0.2 change pernucleotide position. The *numbers at the nodes* indicate the bootstrap values as percentage of 1000 replications. Glucose dehydrogenase enzyme (GI:729809030) from *Escherichia coli* was chosen as the out group for comparison. **a** The phylogenetic position of zlaI protein. **b** The phylogenetic position of zlbI protein. **c** The phylogenetic position of zlar protein. **d** The phylogenetic position of zlbr protein

Web-based similarity searches using the GenBank database were used to determine the homologous degrees of zlaI and zlbI protein sequences to those of other AHL synthases. The most similar homolog of zlaI, identified using a BLASTP search, was an autoinducer synthase from *Sulfitobacter pseuddonitzssschiae* (GenBank accession no. WP 037926640.1) with a 77% identity; the most similar homologs of zlbI were autoinducer synthases from *Tropicibacter naphthalenivorans* and *Ponticoccus* sp. SJ5A-1(GenBank accession nos. WP 058247237.1 and WP 058863594.1, respectively), with 81% identity. In fact, the multiple sequence alignments revealed that zlaI and zlbI share low homology, but both shared 10 conserved amino acids with other reported autoinducer proteins of *Rhodobacterales* bacteria as shown in Additional file 1: Figures S1A, B, respectively. Moreover, the phylogenetic tree constructed based on amino acid alignment (Fig. 3a, b) illustrated that zlaI clustered closely with an autoinducer synthase protein from *Salipiger mucosus* with a bootstrap value of 88% and zlbI was the least phylogenetically related to autoinducer synthase proteins from *T. naphthalenivorans* and *Sagittula stellate* with a bootstrap value of 52%. The Fig. 3c showed that zlaR clustered closely to an luxR family transcriptional regulator protein from *Salipiger mucosus.* Figure 3d showed zlbR clustered closely to luxR family transcriptional regulator proteins from *Pelagibaca abyssi* and *Thiobacimonas profunda* with a bootstrap value of 53%.

### Identification of the zlaI and zlbI genes responsible for AHL production

**Fig. 4:**
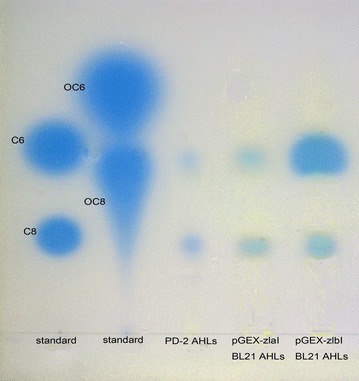
Profiles of AHLs produced by PD-2 and recombinants based on bioautography assay. *Lane A* mixture of synthetic HSL standards: C6 (C6-HSL); C8 (C8-HSL). *Lane B* mixture of synthetic 3-oxo-HSL standards: 3-oxo-C6 (3-oxo-C6-HSL); 3-oxo-C8 (3-oxo-C8-HSL). *Lane C* extracts of strain PD-2. *Lane D* extracts of pGEX-*zlaI E. coli* BL21. *Lane E* extracts of pGEX-*zlbI E. coli* BL21

To identify what kinds of AHLs are produced by the *zlaI* and *zlbI* genes, the expression of *zlaI* and *zlbI* was examined in an *E. coli* strain that does not produce AHL. The presence of the 870 bp *zlaI* and 639 bp *zlbI* genes in the transformants were confirmed by PCR amplification. In bioautography, the KYC55 biosensor detected AHLs from recombinant *E. coli* culture extracts. In Fig. 4, AHLs signals are observed as blue spots on the plat. This demonstrates *zlaI* and *zlbI* encode AHL synthetases in *E. coli* BL21. The profile of AHLs produced by pGEX-*zlaI E. coli* BL21 and pGEX-*zlbI E. coli* BL21 indicate that the *zlaI* and *zlbI* genes produced the same AHLs of 3-oxo-C8-HSL and 3-oxo-C10-HSL (Fig. 4). Although this method cannot determine an absolute quantification, the size of a spot on TLC plate may also reflect the concentrations of AHLs to some degree (Deepa et al. 2012). From the size of each blue spot, the *zlbI* gene produced obviously greater amounts of 3-oxo-C8-HSL than *zlaI*, thereby demonstrating that 3-oxo-C8-HSL is the main product of the *zlbI* gene.

### The effect of β-CD on the growth of strain PD-2

Growth curves with different concentrations of β-CD are shown in Additional file 1: Figure S2. No significant differences were found in the initial 40 h of growth; all the strains entered the logarithmic phase at 10 h and the plateau phase at 40 h. The OD_630_ values declined after 40 h of incubation with β-CD. The levels of growth inhibition were proportional to the β-CD concentrations. At 84 h, the OD_630_ values were 0.67, 0.51, and 0.42 at β-CD concentrations of 1, 2, and 5 mg mL^−1^, respectively. The curve of the control was much more stable (OD_630_ of 0.7) from 40 to 84 h. The results suggested that the addition of 1 mg mL^−1^ β-CD had the lowest impact on growth with a growth of inhibition of only 4%, which we considered appropriate for the following experiments.

**Fig. 5:**
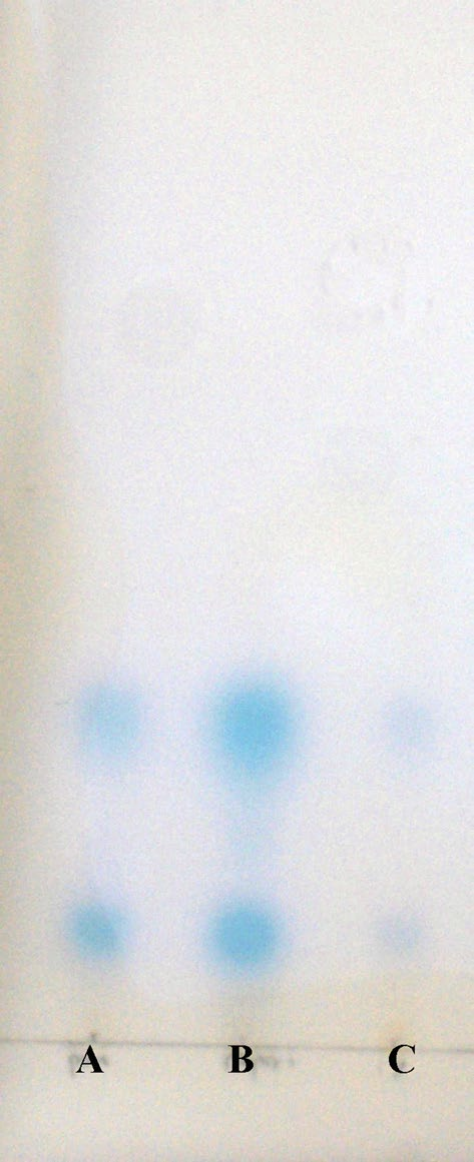
Profiles of AHLs produced by strain PD-2 under different culture condition. The biosensor is *Agrobacterium tumefaciens* KYC55. *Lane A* extracts of strain PD-2 cultured for 20 h. *Lane B* extracts of strain PD-2 cultured for 84 h. *Lane C* extracts of strain PD-2 co-cultured with 1 mg mL^−1^ β-CD at 20 h and continuously cultured for 64 h. After overnight incubation, the AHLs production was visually observed as *blue spots*. There was a positive correlation between the size of the *blue spots* and the concentration of AHLs

After the addition of β-CD, the concentrations of the AHLs were detected by bioautography assay (Fig. 5). The spots of samples A (PD-2 cultured for 20 h at low density) and B (PD-2 cultured for 84 h at high density), which did not contain β-CD, were much larger and were deeper blue than C (A adding β-CD, then cultured for 84 h) which was dim blue. This indicates strain PD-2 at low density just produce a small amount of AHLs. When the bacterium continuously grows to high cell density, the AHLs accumulate to high concentration. But after adding QS-quenching β-CD in the low cell density, the concentration of AHLs do not increase. Because β-CD can form complexes with the AHLs that PD-2 produced at high cell density (Morohoshi et al. 2013).

### The algicidal effect of *Ponticoccus* sp. PD-2 on red tide species

Algicidal activities of strain PD-2 were obtained after 5 days of co-cultivation with target microalgae. The date presented were the mean values of IR from triplicates. (a) The microalgae which was co-cultured with the extracts of strain PD-2 cultured for 84 h. (b) the microalgae which was co-cultured with the extracts of strain PD-2 cultured for 20 h. (c) the microalgae which was co-cultured with extracts of strain PD-2 co-cultured with 1 mg mL^−1^ β-CD on 20 h and continuously cultured for 64 h.

Strain PD-2 shows algicidal effects on all red tide species after 84 h of culturing (Table 1). From the IR value, PD-2 had the strongest algicidal effect (IR 84.81%) on its host, *P. donghaiense*. Besides its host microalgae, strain PD-2 also had algicidal effects on *P. globosa* (IR 79.91%) and a small effect on *A. tamarense* (IR 67.14%). After culturing for 20 h, PD-2 had very little algicidal effects on *P. donghaiense* and *A. tamarense* with IR of 20.27 and 18.38% respectively and no effect on *P. globosa*. The same results were observed when PD-2 was continuously co-cultured with 1 mg mL^−1^ β-CD for 64 h. Then, the algicidal activities of PD-2 co-cultured with β-CD decreased to IR of 19.45% against *P. donghaiense and* 16.09% against *A. tamarense.* Compared to PD-2 without β-CD added, the algicidal activities decrease by more than 50%. In this research, the addition of β-CD reduced the concentration of the AHLs. Thus, the results suggest that the algicidal activities of strain PD-2 are regulated by a QS system.

**Table 1:** Algicidal effect of strain PD-2 against various red tides microalgae species

Inhibition rate (%)	*Prorocentrum donghaiense*	*Phaeocystis globosa*	*Alexandrium tamarense*
A	84.81 ± 2.8	78.91 ± 1.6	67.14 ± 3.5
B	20.27 ± 3.6	−3.61 ± 3.1	18.38 ± 3.3
C	19.45 ± 1.8	−1.94 ± 2.0	16.09 ± 2.4

## Discussion

In this study, a *Ponticoccus* sp. PD-2 strain was isolated from the harmful algal bloom-associated microalga *P. donghaiense*. Its metabolites had growth inhibition effects not only on its host (*P. donghaiense*), but also on two other red tide microalgae, *P. globosa* and *A. tamarense*. *Ponticoccus* was first isolated from the coastal seawaters of Korea and has been identified as a new genus belonging to Rosebacter clade (Hwang and Cho 2008). The *Rosebacter* clade is one of the major marine groups and has members across diverse marine habitats (Buchan et al. 2005). Bacteria of Rosebacter clade also appear in the symbiotic systems of microalgae and may often regulate the algal community (Kodama et al. 2006; Tan et al. 2015).

To our knowledge, this is the first report demonstrating that *Ponticoccus* sp. has QS functions. In our previous research, we found strain PD-2 could produce AHLs QS signals. Of the diverse AHLs produced by marine bacteria, the most common types are C4-HSL, C6-HSL, 3-hydroxy-C6-HSL, 3-oxo-C6-HSL, C8-HSL, 3-oxo-C8-HSL, 3-oxo-C10-HSL, 3-oxo-c12-HSL and C14-HSL (Berger et al. 2012; Swift et al. 1997; Tang et al. 2013; Zan et al. 2013). The various signal molecules produced by its synthetases (*luxI* homolog genes) can be investigated by heterologous expression (Gao et al. 2014; How et al. 2015; Morohoshi et al. 2016). To determine which kinds of AHLs are produced by *zlaI* and *zlbI*, we expressed *zlaI* and *zlbI* in *E. coli*. 3-oxo-C8-HSL and 3-oxo-C10-HSL were identified in a PD-2 extract and in *E. coli* overexpressing *zlaI* and *zlbI*, suggesting that both *zlaI* and *zlbI* can each produce two kinds of AHLs. However, it is rare that two homologous *luxI* genes can generate identical signal molecules in one strain. Thus, we speculate that the two kinds of signal molecule are synthesized from the same metabolic pathway. In this study, the multiple sequence alignment and the phylogenetic tree (Additional file 1: Figure S1; Fig. 3a, b) illustrated that although there was only a low degree of homology, both of PD-2 and other *Rhodobacterales* bacteria had conserved regions among AHL synthases. Thus, the *zlaI* gene and *zlbI* gene should be new AHL synthetase genes. All the strains shared the 10 invariant amino acids that are characteristic of *luxI* homologs (Parsek et al. 1997). This strongly indicates a low rate of random mutation for this autoinducer. It also shows that these proteobacteria share a similar QS mechanism and a similar mechanism for regulating the synthesis of AHLs, although the corresponding AHLs regulate different target genes. Through comparing zlaR and zlbR protein sequences with those of other LuxR family transcriptional regulator on NCBI and constructing phylogenetic tree (Fig. 3c, d), we found that there was also a low degree of homology among transcriptional regulator from other bacteria. Thus, the *zlaR* gene and *zlbR* gene might also be new transcriptional regulator genes.

Many studies have shown that marine organism-associated bacteria play very important roles when interacting with their hosts. In recent years, a high percentage of marine organism-associated bacteria have been shown to produce QS signals, including bacteria associated with microalgae, sponges, corals, and fish (Britstein et al. 2016; Wagner-Döbler et al. 2005). Thus, studying the QS-mediated interactions between marine bacterial with their hosts has profound theoretical and practical significance regarding the ecological functions of marine bacteria. In fact, many investigations have proven that QS regulates such interactions. The luminous bacterium *Vibrio fischeri* is the specific light organ symbiont of *Euprymna scolopes* (Boettcher and Ruby 1990) *E. scolopes* is a small sepiolid squid (average adult length, 40 mm) that is indigenous to the Hawaiian archipelago, where it lives in shallow sand flats that are associated with coral reefs (Ruby and McFallngai 1992). The squid buries itself in the sand during the day to escape predators, but it comes out to forage in the water column at night (Summers 1983). Additionally, sponges and their associated microorganisms have developed a variety of chemical defense pathways. The production of chemicals which are regulated via QS systems are employed to defend sponges against potentially pathogenic bacteria and fish predators, deter fouling organisms, and degrade surfactants (Guo et al. 2011; Huang and Li 2006).

Cyclodextrins are a family of compounds comprising sugar molecules that are bound together in a ring. Cyclodextrins are well known cyclic oligosaccharides that form complexes with a wide range of organic compounds. Tsukasa proved that α,β-CD could form a complex with an autoinducer in a bacterial culture medium (Ikeda et al. 2002). He found that the addition of β-CD decreased the abundance of AHLs signal molecules by 75%, without inhibiting bacterial growth. But our results indicate that 1 mg mL^−1^ β-CD had little influence on the growth of strain PD-2, and β-CD could form a complex with AHLs produced by strain PD-2 in the culture medium (Morohoshi et al. 2013). The algicidal activities of strain PD-2 decreased more than 50% due to its QS system was inhibited, this is the first evidence that the QS systems of microalgae-associated bacteria can regulate algicidal activity.

Existing evidence proves that production of algicidal substances can be regulated by QS (Guo et al. 2016; Nakashima et al. 2006), although there is no direct evidence to suggest that the algicidal activities of microalgae-associated bacteria is regulated by QS systems. Consequently, investigations of the effects of QS on the regulation of algicidal functions are needed to increase our understanding of the relationship between bacteria and algae, and QS inhibitors can be used to study QS regulation.

## Additional file

**Additional file 1.** Additional figures.

## Abbreviations

QS: quorum sensing
AI: autoinducer
AHL: *N*-acyl-homoserine lactone
β-CD: β-cyclodextrin
GC–MS: gas chromatography–mass spectrometer
HPLC–MS: high-performance liquid chromatography-mass spectrometer
LB: Luria-Bertani
TLC: thin-layer chromatography
IR: inhibition rate
RF: retardation factor
3-oxo-C8-HSL: *N*-3-oxo-octanoyl-L-homoserine lactone
3-oxo-C10-HSL: *N*-(3-oxodecanoyl)-L-homoserine lactone
RT: retention times
PCR: polymerase chain reaction
CCMM: culture collection of marine microalgae
