# Supplementary material for: Quorum sensing of microalgae associated marine Ponticoccus sp. PD-2 and its algicidal function regulation
Source: AMB Express. 2017 Mar 9;7:59. doi: 10.1186/s13568-017-0357-6 (PMC5344870; doi:10.1186/s13568-017-0357-6)
Supplement: Supplementary file 1 — Additional file 1. Additional figures. [file 13568_2017_357_MOESM1_ESM.doc]

**Quorum Sensing of Microalgae Associated Marine *Ponticoccus* sp. PD-2 and Its Algicidal Function Regulation**

Wendan Chi^a^, Li Zheng^a,b*^, Changfei He^a^, Bin Han^a^, Minggang Zheng^a^, Wei Gao^a^, Chengjun Sun^a,c^, Gefei Zhou^d^, Xiangxing Gao^e^

^a^Key laboratory for marine bioactive substances and modern analytical technology of the First Institute of Oceanography, State Oceanic Administration, Qingdao, 266061, China

^b^Laboratory for Marine Ecology and Environmental Science, Qingdao National Laboratory for Marine Science and Technology, Qingdao, 266071, China

^c^Laboratory for Marine Ecology and Environmental Science, Qingdao National Laboratory for Marine Science and Technology, Qingdao, 266071, China

^d^School of Life Science of Yantai University, Yantai, 264000, China

^e^National Deep Sea Center, State Oceanic Administration of China, Qingdao, 266237, China

^*^Corresponding author

Mailing Address: the First Institute of Oceanography, State Oceanic Administration, No. 6 Xianxialing Road, Qingdao, Shandong Province, 266061, PR China

Phone: (86) 532-88961802

Fax: (86) 532-88963253

E-mail: zhengli@fio.org.cn

***Running title:*** Quorum Sensing and Algicidal Function

A


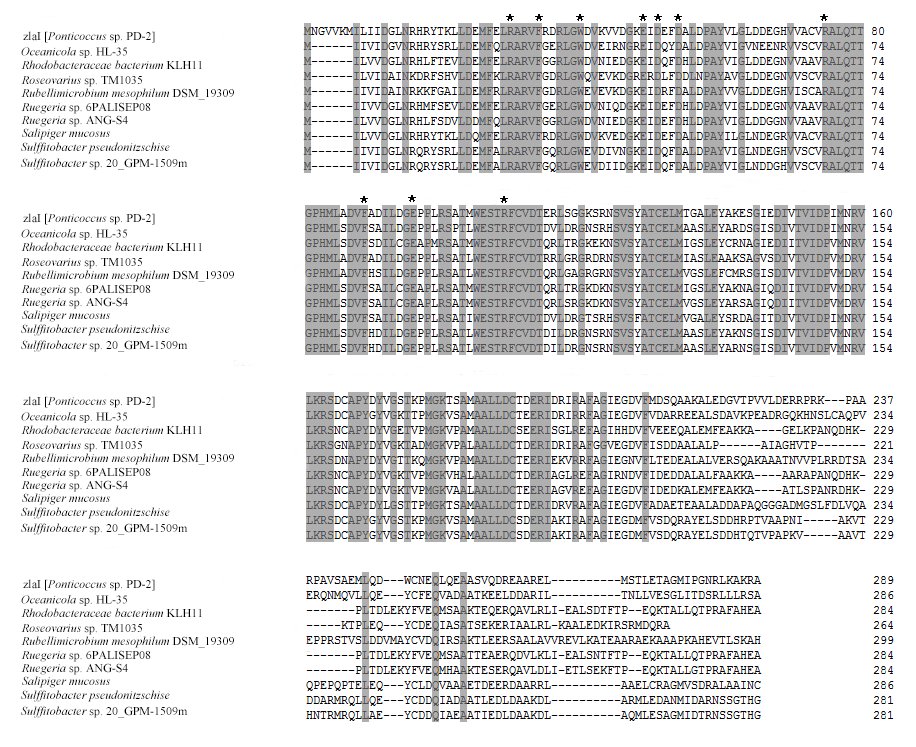


B


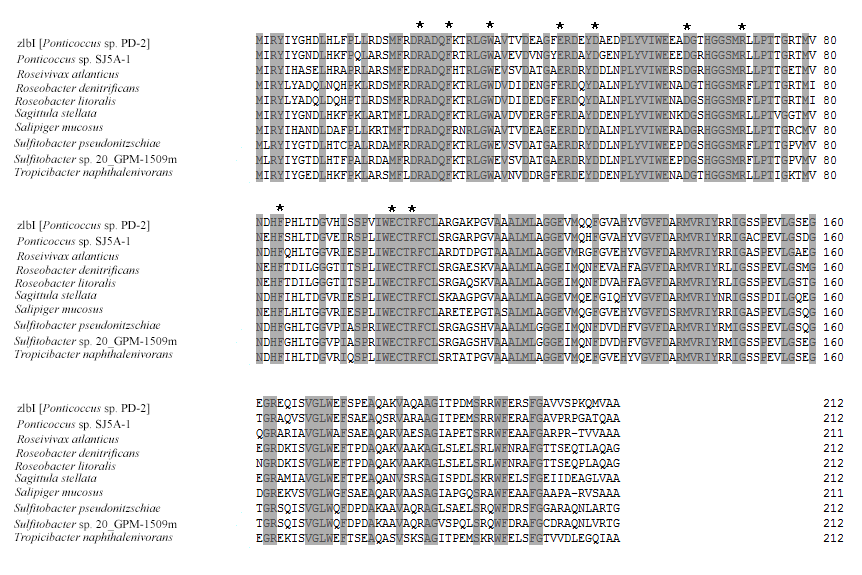


Fig.S1 Multiple sequence alignment of *N*-acylhomoserine lactone (AHL) autoinducer protein sequences of strain PD-2 with other protein sequences. Sequences were derived from NCBI database (http: //www.ncbi.nlm. nih.gov) and were aligned using DNASTAR.Lasergene.v7.1 software. Residues that are identical among the sequences were given a gray background. The 10 invariant amino acids characteristics of *luxI* homologues were denoted with asterisks. GenBank accession numbers (inparentheses): **(A)** autoinducer synthesis (zlaI) from *Ponticoccus* sp. PD-2 (WP_031323077.1), autoinducer synthesis from *Oceanicola* sp. HL-35 (WP_024811268.1), autoinducer synthesis from *Rhodobacteraceae* bacterium KLH11 (WP_008757604.1), autoinducer synthesis from *Roseovarius* sp. TM1035 (WP_008282297.1), autoinducer synthesis from *Rubellimicrobium mesophilum* DSM_19309 (EYD73947.1), autoinducer synthesis from *Ruegeria* sp. 6PALISEP08 (WP_050602108.1), aoutoinducer synthesis from *Ruegeria* sp. ANG-S4 (WP_039529872.1), autoinducer synthesis from *Salipiger mucosus* (WP_020039175.1), autoinducer synthesis e from *Sulffitobacter pseudonitzschise* (WP_037926640.1), autoinducer synthesis from *Sulffitobacter* sp. 20_GPM-1509m (WP_028955112.1) **(B)** autoinducer synthesis (zlbI) from *Ponticoccus* sp. PD-2 (WP_039616214.1), autoinducer synthesis protein from *Ponticoccus* sp. SJ5A-1 ( WP_058863594.1), autoinducer synthesis protein from *Roseivivax atlanticus* (WP_043842873.1), autoinducer synthesis protein from *Roseobacter denitrificans* (WP_011567886.1), autoinducer synthesis protein from *Roseobacter litoralis* (WP_013962934.1), autoinducer synthesis protein from *Sagittula stellate* (WP_005858901.1), autoinducer synthesis protein from *Salipiger mucosus* ( WP_020038895.1), autoinducer synthesis protein from *Sulfitobacter pseudonitzschiae* ( WP_037925144.1), autoinducer synthesis protein from *Sulfitobacter* sp. 20_GPM-1509m (WP_028954835.1), autoinducer synthesis protein from *Tropicibacter naphthalenivoran*s (WP_058247237.1)

Fig.S2 Growth curves of strain PD-2 under different β-CD concentrations. The cell density measured via absorbance at OD_630_. ■: control, PD-2 without β-CD. ▲,▼,●: present strain PD-2 co-cultured with 1, 2, 5 mg mL^-1^ β-CD respectively.
